# Numerical Analysis of the Dynamic Properties of Bionic Raster Ceilings

**DOI:** 10.3390/ma17163925

**Published:** 2024-08-07

**Authors:** Artur Wirowski, Ewelina Kubacka, Paulina Kaszubska, Weronika Walisiak

**Affiliations:** 1 Department of Structural Mechanics, Lodz University of Technology, 93-590 Lodz, Poland; artur.wirowski@p.lodz.pl; 2 Faculty of Civil Engineering, Architecture and Environmental Engineering, Lodz University of Technology, 93-590 Lodz, Poland; paulina.kaszubska01@gmail.com (P.K.); 248883@edu.p.lodz.pl (W.W.)

**Keywords:** dynamic analysis, frequency, airflow, ceiling panels, bionic, finite element method

## Abstract

In this study, a numerical dynamic analysis of ceiling raster panels was performed. The analysis was conducted on panels designed with inspiration from bionics. The purpose of the analysis was to enable optimisation of the location of the holes in the designed slabs in order to achieve the preferred dynamic properties, including the natural frequencies of the slabs and an appropriate airflow to avoid the occurrence of resonance. Three different types of panels were used and a total of fifteen panels were designed in terms of their geometry, with circular, elliptical, and hexagonal perforations, made of different materials: polypropylene PP, wood, and aluminium. Then, using the finite element method and ANSYS 2023 R1 software, the airflow over the ceiling panels and their natural frequencies and vibration modes were analysed. The analysis took into account not only the shape of the openings, but also their percentage area relative to the total panel area and different airflow velocities. In addition, the results were compared in an analytical way with those obtained for a solid slab. The results obtained include findings on the mode shapes and values of the vibration frequencies of the plates, air pressure maps, histograms, and plots of the pressure dependence on the surface area of the plate openings.

## 1. Introduction

In the face of growing awareness of the thermal comfort [1] of occupants, which has a significant impact on their well-being, work efficiency, and sense of fatigue, an adequate ventilation system to ensure good air quality is becoming one of the basic installations in buildings. Such systems play a particular role in public buildings, schools, and universities. For many years, the focus has not only been on ensuring an appropriate indoor temperature, but also on analysing the distribution of this temperature and air velocity in order to eliminate local draughts and significant vertical air temperature differences. For this purpose, air conditioner blowers in different locations [2], the presence of various equipment in the room under consideration, or the geometry of air inlets and outlets, have been considered [3].

Nowadays, one of the more innovative developments in HVAC (heating, ventilation, and air conditioning) systems, the diffuse ceiling ventilation system [4], can solve the problems associated with uneven airflow inside a room. In this system, fresh air is supplied into the space between the suspended ceiling and the slab, called the plenum [5], and then distributed around the room through so-called active panels made of permeable porous materials or non-porous but perforated materials, due to the pressure difference between the plenum and the ventilated space. The main advantages of this system include the elimination of draughts and a high cooling capacity compared to conventional ventilation systems. This means that distributed air conditioning is able to remove more heat from the space, while ensuring the thermal comfort of the occupants [6]. In addition, it is worth noting that both active and passive panels can be shaped from different materials and can have different shapes, depending on the expectations of the users or the purpose of the space. Due to their porous structure, raster ceilings enable the use of diffuse air conditioning in rooms. The bionic-inspired [7] raster ceilings analysed in this thesis are a response to the growing expectations of consumers, who are increasingly rejecting simple, repetitive, austere shapes in favour of the use of ecological, modern, interesting solutions that create architectural interiors full of soothing patterns inspired by nature. Borrowing patterns from the surrounding natural environment aims to ensure that the occupants of a given space feel less stressed and that their creativity increases.

The primary purpose of ventilation systems is to provide a building with a constant supply of fresh air at the correct temperature and to remove stale air and pollutants. However, this study addresses the issue of acoustic comfort [8], which is another crucial aspect when designing spaces for effective and creative work or study. Flowing air and operating fans and air conditioners can cause vibrations in raster ceilings, thereby generating unnecessary noise, leading to annoyance, irritation, and difficulties in maintaining full concentration for the occupants. Surveys and studies indicate that sound propagation from mechanical air-conditioning systems has a negative impact on speech perception, language skills, or episodic memory [9]. Depending on the difference between the user’s sound level and the noise (signal-to-noise ratio, SNR), a reduction in users’ concentration levels, a significant degradation in test performance, and an extension of reaction times with increasing noise levels, have been reported [10].

To ensure acoustic comfort in work and study spaces, the dynamic properties of the analysed ceilings are essential, defining their behaviour when subjected to time-varying influences generated by airflow and fan operation. The most important dynamic properties, which allow for predicting ceiling behaviour and reducing vibrations, are natural vibration frequencies and vibration modes. These properties depend on a series of factors such as the material of the panels, their shape and dimensions, as well as the surface area of the airflow openings, their geometry and arrangement.

The aim of this study is to conduct an extensive numerical analysis of the dynamic properties of bionic-inspired, suspended raster ceilings. This analysis will enable future optimisation of the distribution of the perforations in the ceiling design so that, while maintaining the user-friendly appearance of the ceiling, the preferred dynamic properties can be achieved: adequate airflow and natural frequencies of the structure that do not generate resonance phenomena during airflow through the ceiling.

Fifteen different panel geometries were designed to perform the analysis described above. Each of the plates was analysed numerically in terms of its natural frequencies and airflow to show the differences in the behaviour of the panels made of different materials and with different surface areas and opening locations. The ANSYS software was used for the analyses.

Carrying out such analyses allows not only the design of raster ceiling panels with any chosen materials, but also the selection of the appropriate dimensions or thicknesses. Designing the appropriate arrangement of openings of different shapes and sizes affects not only the aesthetic qualities, but also the insulating and acoustic properties, and this in turn allows the reduction of vibrations and noise and, consequently, benefits the wider comfort of the user. The variants of the designed panels, which are optimally selected due to the adopted criterion, can be introduced to the market in the future in cooperation with the relevant industries.

## 2. Problem Analysis

### 2.1. Geometry and Materials of Analysed Plates

The geometry of the analysed panels was influenced both by aesthetic aspects and bionics, as well as engineering and executive considerations. At the same time, the proposed and analysed opening geometries had to be varied in terms of size and location, so that the obtained results could be analysed statistically.

Therefore, it was decided to keep the square dimension of the panel area constant in each analysed case (0.48 m), thus the surface area of the analysed plates was equal to 0.23 m^2^. This size is based on typical engineering applications for raster ceiling panels. The area of the panel is usually of a modular size (0.5 m), while the panel itself has to be slightly smaller in order to be fixed freely to the frame.

**Figure 1:**
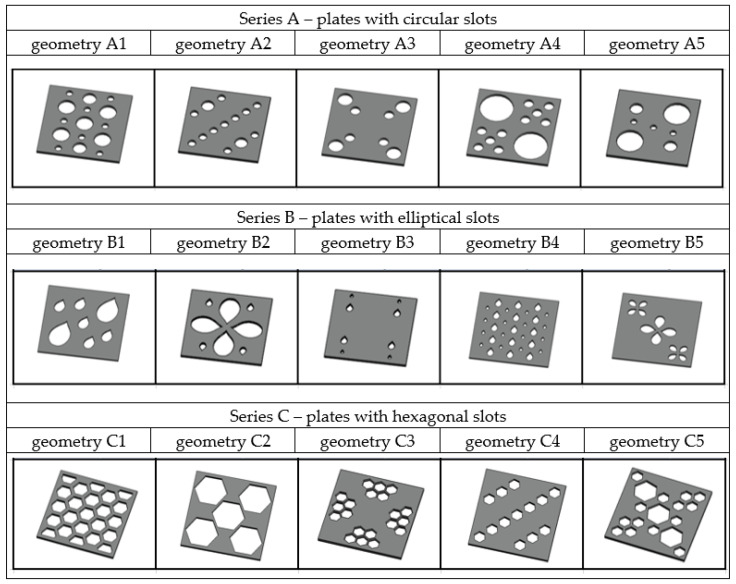
Geometry of analysed plates.

The shapes of the holes were created based on inspiration from bionics, while their arrangement was proposed using aesthetic aspects and various symmetries. In this way, three series of plates were created with different perforation shapes (A—circular, B—elliptical, C—hexagonal). In each series, five plates were designed and are shown in Figure 1. The areas of the holes and the values of the first- and second-order ‘pseudostatic’ moments in terms of the geometry of each plate are presented in Table 1. The first-order pseudostatic moment is defined as the product of the areas of the individual openings (A_i_) and their distance from the centre of gravity of the panel (d_i_), while the second-order pseudostatic moment is defined as the product of the areas of the individual openings and the square of their distance from the centre of gravity of the panel.

Each series of panels was modelled with a different material so that the dynamic properties of the type of structure could be investigated regardless of the type of material used and the stiffness of the panel. The material properties and thickness of the individual plates are shown in Table 2.

The aforementioned ceiling panel materials were selected due to their different material properties and ecological aspects; wood is a naturally occurring material that is environmentally friendly, while having a porous structure that absorbs sound [11]. Aluminium is the base material, chosen because of the ubiquity of panels made from this material. Polypropylene, on the other hand, is included because of the significance of the development in the polymer industry (since polypropylene belongs to the polyolefin group and is obtained by polymerising propene). Polymers have become one of the basic materials for structural and finishing elements in the construction industry, along with wood, metals, concrete, or glass, due to their durability and versatility [12].

### 2.2. Research Methods Used

To verify the accuracy of the numerical models, as well as to make a preliminary determination of the theoretical relationships between the model parameters, theoretical analysis of the natural frequencies of solid square plates was conducted. It was assumed that the analysed slab was an isotropic Kirchhoff thin plate [13] and is, therefore, described by the following equation:

(1) $$D{\text{∇}}^{2}{\text{∇}}^{2}w=q\left(x,t\right)\;-\;\rho h\ddot{w},$$

where q(x,t) is the external load, h is the thickness of the plate, w is the deflection of the centre plane of the plate, ρ is the density per unit area, and D is the bending stiffness of the plate, which is expressed by the following formula:

(2) $$D=Eh^{3}{[12\left(1\;-\;\upsilon^{2}\right)]}^{-1},$$

where E is Young’s modulus and υ is Poisson’s ratio. When estimating the natural vibration of a thin plate, it was assumed that there was no external load and also that the vibrations occurred harmonically, in the following form:

(3) $$w\left(x_{1},x_{2},t\right)=W\left(x_{1},x_{2}\right)F(t),$$

where F(t) is expressed as follows:

(4) $$F\left(t\right)=Ae^{i\omega t}+Be^{-i\omega t}.$$

where A and B are the integration constants, ω is the natural vibration frequency, and t is the time coordinate.

Assuming simply that support conditions in the midplane of the plate are present at all four edges, it is assumed that:

(5) $$w_{\mathrm{mn}}\left(x_{1},x_{2}\right)=\sin(m\pi x_{1}a^{-1})\sin(n\pi x_{1}a^{-1}),$$

where w_mn_ describes the mode shape, m and n are the wave numbers in two directions, while a is the dimension of the plate. Ultimately, the vibration frequencies were obtained as:

(6) $$\omega_{\mathrm{mn}}=\pi^{2}\left(m^{2}+n^{2}\right)a^{-2}{\left(D\rho^{-1}\right)}^{1/2}.$$

**Figure 2:**
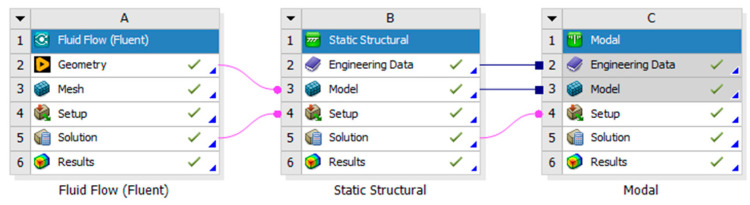
Diagram of module connections in the ANSYS program.

The numerical analysis was carried out using ANSYS 2023 software. The analysis consisted of three stages: simulation of the airflow through the slab using computational fluid dynamics (CFDs), static analysis of the deformations and stresses under the influence of self-weight and air pressure, and modal analysis to determine the dynamic properties of the slabs [14]. The appropriate combination of modules, shown in Figure 2, allowed commonality in terms of the geometry, material properties, and import of loads during the different stages.

The airflow analysis was based on a turbulent model as part of the Reynolds-averaged Navier–Stokes (RANS) approach using the Boussinesq hypothesis [15], which assumes the expression of the Reynolds turbulent stress as a function of the mean strain rate tensor, according to the following formula:

(7) $$\tau_{\mathrm{ij}}=\;-2\mu_{t}\left[S_{\mathrm{ij}}-\frac{1}{3}\frac{\text{∂}U_{k}}{\text{∂}x_{k}}\delta_{\mathrm{ij}}\right]-\frac{2}{3}\rho k\delta_{\mathrm{ij}},$$

where μt is the turbulent viscosity; ρ is the density; δij is the Kronecker delta; i, j are indicial notations with Einstein’s summation convention and take values 1, 2, 3 (refers to all equations presented in this paper); U denotes velocity; and Sij is the mean stress rate tensor defined as:

(8) $$S_{\mathrm{ij}}=\frac{1}{2}\left[\frac{\text{∂}U_{i}}{\text{∂}x_{j}}+\frac{\text{∂}U_{j}}{\text{∂}x_{i}}\right],$$

And k is the turbulent kinetic energy expressed as half of the sum of the variances of the velocity components, a follows:

(9) $$k=\frac{1}{2}\overset{-}{u_{i}\text{’}u_{i}\text{’}},$$

where ui′ is the difference between the instantaneous and average velocity.

One of the most popular turbulent models, the standard k-ε model, whose equations were derived in 1972 by Jones and Laudner [16], was used to calculate the turbulent viscosity μt, which depends on, among other things, the fluid properties, turbulence intensity, solid shape, and boundary conditions. The chosen k-ε two-equation turbulence model, which employs the Boussinesq hypothesis on the proportionality of Reynolds stresses to the strain rates, is essentially the simplest complete turbulence model used to predict the properties of a given turbulent flow without previous knowledge of its structure. It is also one of the most popular turbulence models used by both engineers and researchers and is available in most CFDs analysis software. The transport equations for turbulent kinetic energy k and the turbulent dissipation rate ε were formulated as follows [17]:

(10) $$\frac{\text{∂}\left(\rho k\right)}{\text{∂}t}+\frac{\text{∂}\left(\rho U_{j}\varepsilon \right)}{\text{∂}x_{j}}=\tau_{\mathrm{ij}}\frac{\text{∂}U_{i}}{\text{∂}x_{j}}-\rho \varepsilon \;+\;\frac{\text{∂}}{\text{∂}x_{j}}\left[\left(\mu \;+\;\frac{\mu_{t}}{\sigma_{k}}\right)\frac{\text{∂}k}{\text{∂}x_{j}}\right],$$

(11) $$\frac{\text{∂}\left(\rho \varepsilon \right)}{\text{∂}t}+\frac{\text{∂}\left(\rho U_{j}\varepsilon \right)}{\text{∂}x_{j}}=C_{\varepsilon 1}\frac{\varepsilon }{k}\tau_{\mathrm{ij}}\frac{\text{∂}U_{i}}{\text{∂}x_{j}}-C_{\varepsilon 2}\rho \frac{\varepsilon^{2}}{k}+\frac{\text{∂}}{\text{∂}x_{j}}\left[\left(\mu \;+\;\frac{\mu_{t}}{\sigma_{\varepsilon }}\right)\frac{\text{∂}\varepsilon }{\text{∂}x_{j}}\right],$$

where μ is the dynamic molecular viscosity and μ_t_ is the turbulent viscosity, expressed by the following formula:

(12) $$\mu_{t}=c_{\mu }\frac{k^{2}}{\varepsilon },$$

The constants appearing in the above equations were adopted according to the modification introduced by Launder and Scharm in 1974 [18] as: Cε_1_ = 1.44, Cε_2_ = 1.92, Cμ = 0.09, σ_k_ = 1.00, and σ_ε_ = 1.00 [19].

**Figure 3:**
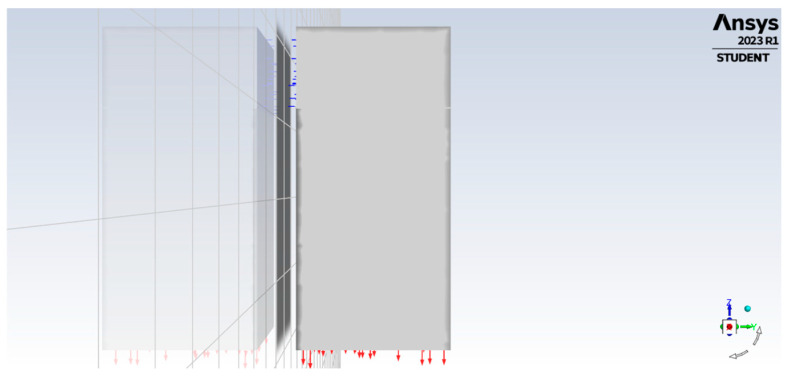
Location of air inlet and outlet.

The air inlet was modelled along the direction of the positive y-axis, while the outlet was 0.75 m below the bottom surface of the slab, as shown in Figure 3. Three different air velocities were analysed at the inlet: 1.5 m/s, 3.0 m/s, and 10.0 m/s. The outer walls of the airspace were assumed to be non-slip.

**Figure 4:**
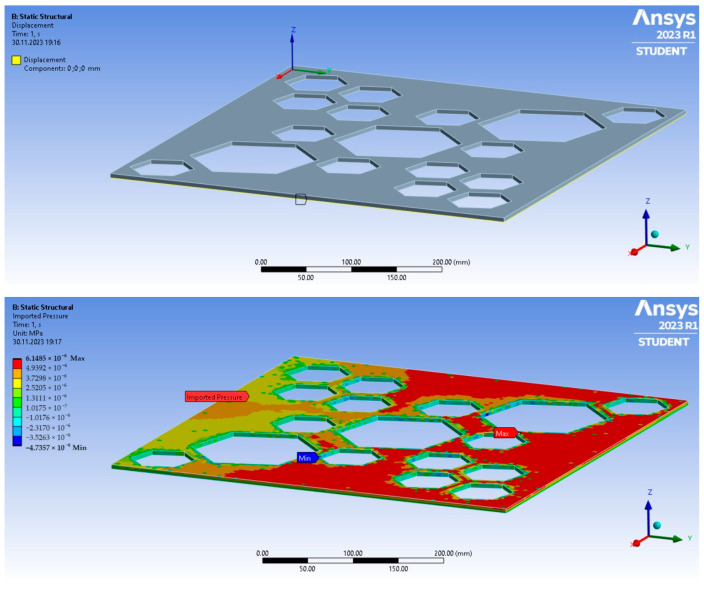
Boundary conditions for the static calculation (**top**) and a view of the imported pressure from the flow analysis (**bottom**).

To analyse the deformations and stresses on the plates under the pressure caused by the airflow, the Static Structural module was used. The slabs were modelled as simply supported; the displacement in the x-, y-, and z-axis directions was blocked at the bottom outer edges, as shown in Figure 4. Due to the connection with the module responsible for flow simulation, the pressure was automatically included in the calculation over the entire surface of the slab (cf. Figure 4).

The dynamic analysis of the plates was conducted by adopting the pre-stress parameters from the static analysis, including the support conditions and loads. The first six vibration frequencies of the structures and their corresponding mode shapes were determined, as well as the directional deformations along the x, y, and z axes.

**Figure 5:**
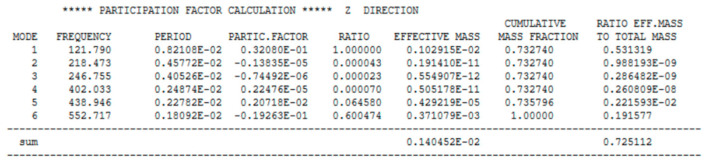
Example of participation factor calculation for displacement along the z-axis.

In addition, the results were obtained in the form of ‘participation factor calculations’ [20], calculated for all six degrees of freedom, indicating the contribution of a given displacement on the deformation for a given vibration frequency and, therefore, allowing a better understanding of the dynamic response of the slab to the interactions, cf. Figure 5.

The dynamic analysis was limited to the effect of the static flow velocity pressure. The influence of possible dynamic flow effects on the dynamic behaviour of the plates (e.g., fluttering) was omitted.

## 3. Results

### 3.1. Flow Analysis

The flow analysis focused on parameters that are most significant for the loading of the slabs and the thermal and acoustic comfort of the space’s users, i.e., the pressure exerted by the airflow on the plate’s surface and the velocities and directions of this flow under the plate.

**Figure 6:**
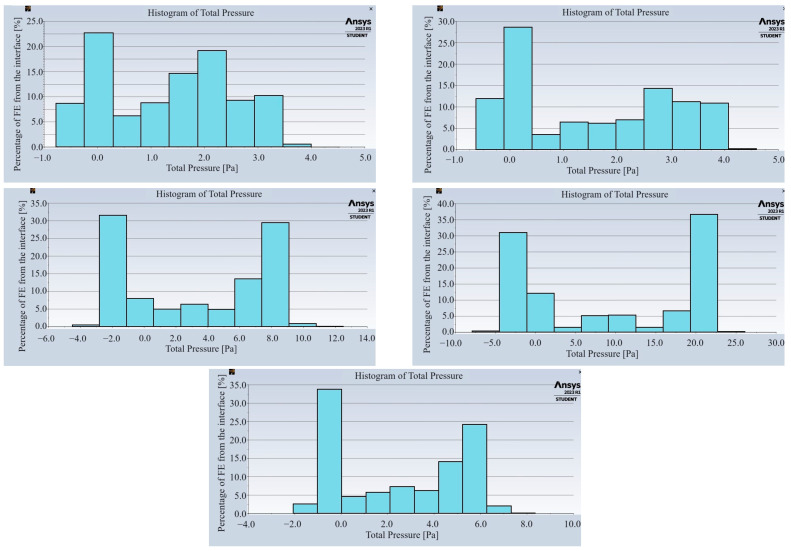
Histograms of pressure distribution on plates C1–C5, with a thickness equal to 5 mm (horizontally from left), for a velocity of 1.5 m/s.

The pressure values at the air–slab interface are strongly dependent on the area of the openings in the slab and their location. The histograms presented in Figure 6 show the percentage of the finite elements from the interface, for which the total pressure value falls within the range described on the horizontal axis.

**Figure 7:**
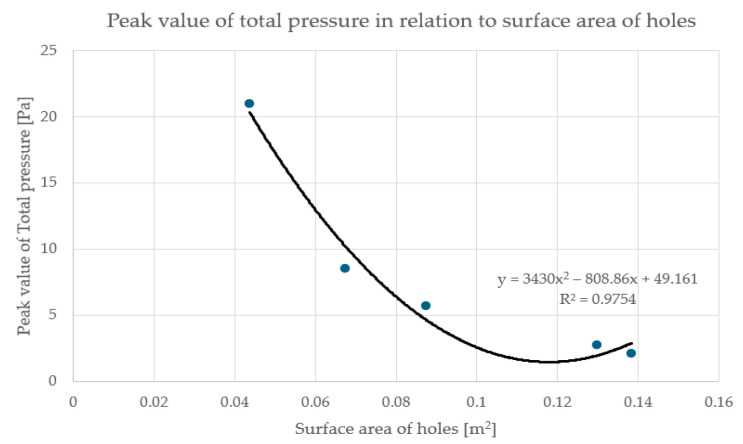
Dependence of the positive pressure value peak on the surface area of the openings.

Each of the histograms shown in Figure 6 has an initial peak at a pressure of around 0 Pa or slightly below 0 Pa, which corresponds primarily to the contact surface between the underside of the slab and the air beneath it. The next peak corresponds to a positive pressure value, which varies strongly depending on the surface area of the openings in the panel. For plate C1 (percentage of holes 60%), the peak occurs at around 2 Pa, while for plate C4 (percentage of holes 19%) it occurs at around 20 Pa, for the same inlet air velocity of 1.5 m/s. Therefore, it can be concluded that the smaller surface area of the openings in the slab, the higher the value of the pressure caused by the airflow acting on the plate, cf. Figure 7. In this figure, the horizontal axis shows the area of the holes in the plate and the vertical axis shows the positive pressure value corresponding to the peak in the histogram. This relationship can be approximated by a second-degree polynomial equation.

**Figure 8:**
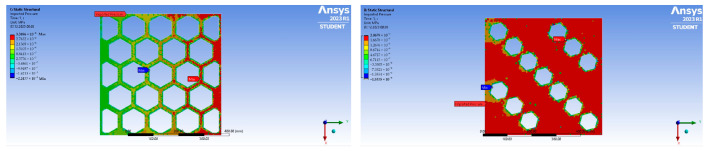
Distribution of imported pressure on plates C1 (**left**) and C4 (**right**) for a velocity of 1.5 m/s.

In addition, it should be noted that the peaks of the plates with a smaller opening surface area are more pronounced than for plates with a large opening surface area, which means that the pressure distribution on the plates with lower porosity is more uniform. This relationship is clearly visualised by the pressure maps imported for the static calculation and dynamic analysis (cf. Figure 8).

**Figure 9:**
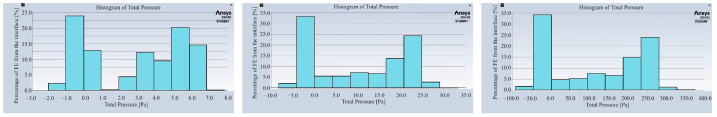
Histograms of pressure values at the air–plate interface for plate C5, with a thickness equal to 2 mm, for velocities of 1.5, 3.0, and 10.0 m/s (from left to right).

The pressure value acting on the plate is, of course, strongly dependent on the inlet air velocity. However, changes in velocity have a much greater impact on the pressure value than on its distribution, especially at higher velocities, as illustrated by the histograms for plate C5 (cf. Figure 9).

**Figure 10:**
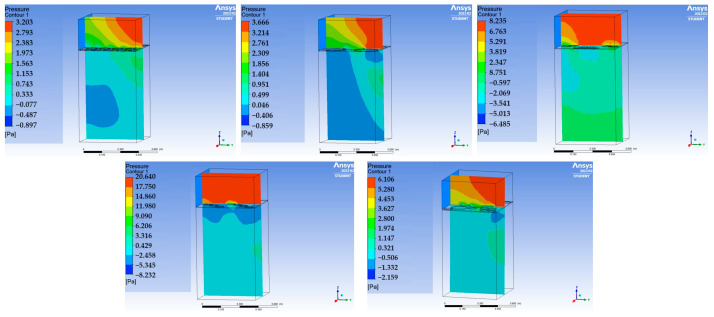
Pressure maps in regard to the plane perpendicular to the inlet plane (left inlet) at the mid-span of plates C1–C5 (horizontally from left), for an inlet air velocity of 1.5 m/s.

The pressure distribution in the space between the plate and the slab (known as the plenum) is also strongly dependent on the surface area and distribution of the openings in the plate. Based on the pressure maps presented in Figure 10, it can be observed that for plates with openings with a large surface area (C1, C2), there is a greater pressure difference in the air within the plenum. Consequently, the air moves more quickly (i.e., closer to the inlet) and less uniformly through the openings into the space beneath the plate.

In contrast, the smaller the surface area of the openings in a slab, the less variable the pressure above it, thus the influence of a short plenum is reduced and local draughts are eliminated. This indicates the validity of using slabs with openings with a smaller surface area, in particular in the immediate vicinity of air conditioners and fans, in order to ensure the thermal comfort of the users of spaces located close to the source of the air inflow.

**Figure 11:**
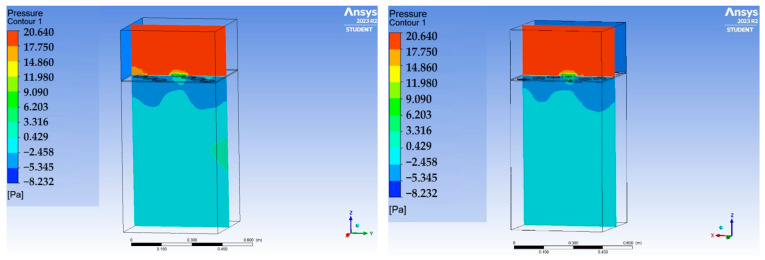
Pressure map for plate C4 in the plane perpendicular to the inlet (**left**) and in the plane parallel to the inlet (**right**) for an inlet air velocity of 1.5 m/s.

In addition, analysing the pressure maps in the plane parallel to the inlet, it can be seen that for panels with a small distribution field of openings, almost complete symmetry of the pressure distribution in both directions is obtained (Figure 11).

The natural consequence of the reduction in the airflow area relative to the inlet area is an increase in air velocity as it flows through the plate. The numerical analyses conducted confirms this relationship. The smaller the area of the perforations in the slab, the greater the observed increase in flow velocity.

**Figure 12:**
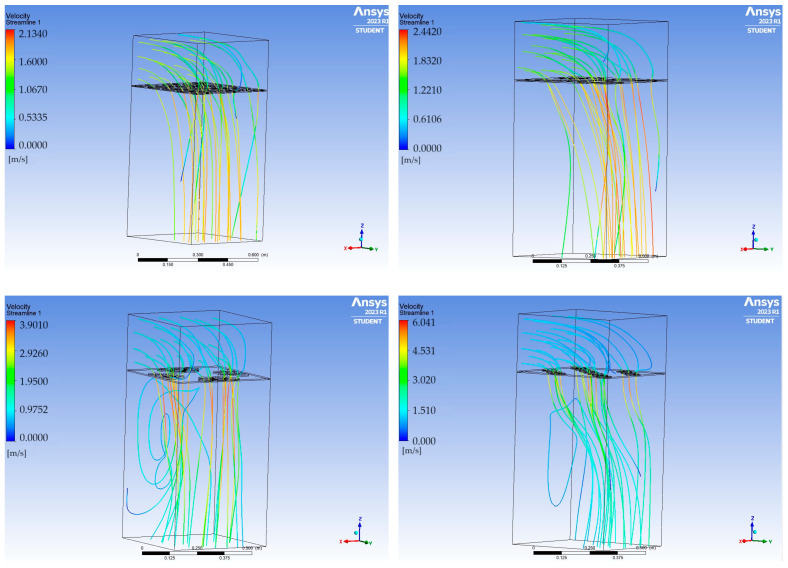
Streamline for panels C1–C4 (horizontally from left) for an inlet air velocity of 1.5 m/s.

The manner of airflow, its direction, local vortices, or accelerations, depends on the distribution of the openings. For evenly distributed openings (plates C1, C2) with a large total surface area, the flow in the space under the plate is calm, without sudden changes in direction or speed, although it is strongly concentrated in the part of the space under the plate located furthest from the inlet. In contrast, for plates with an irregular distribution of openings, particularly with blocked flow in the middle of the span, as in plate C3, local flow vortices, changes in direction, and changes in velocity can be observed. A similar phenomenon, although on a smaller scale, can be seen for plate C4. Blocking too large an area of flow can, therefore, result in a more chaotic flow, which is also more difficult to model and predict (Figure 12).

### 3.2. Dynamic and Static Analysis of the Plates

**Figure 13:**
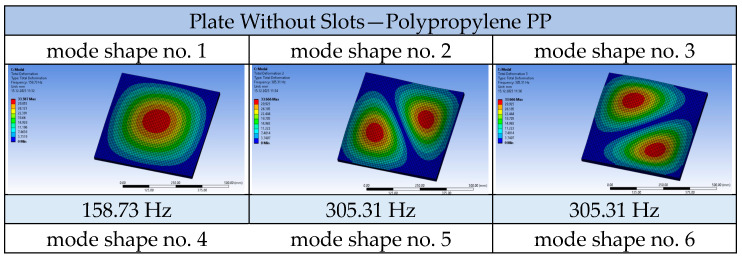

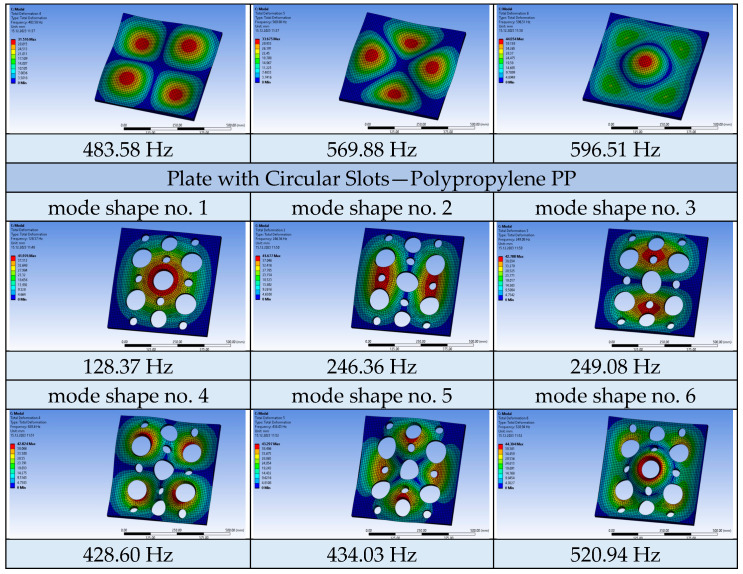
Mode shapes and frequencies for the plates made of polypropylene (PP) at a flow velocity of 1.5 m/s.

**Figure 14:**
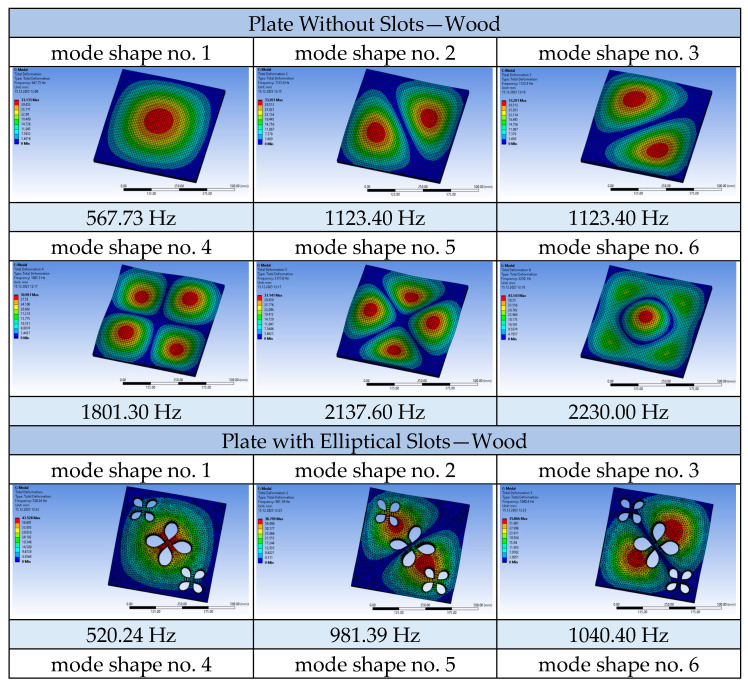

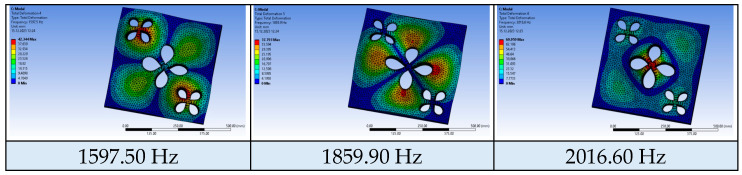
Mode shapes and frequencies for the plates made of wood at a flow velocity of 1.5 m/s.

Using the ANSYS 2023 R1 software, a modal analysis of the tested plates was conducted, obtaining the frequencies and mode shapes for each of them. Calculations were also performed for a solid plate made of each material used, namely aluminium, wood, and polypropylene, as well as for each of the three tested airflow velocities. Selected results are presented in the following tables: solid plate and plate with circular openings made of polypropylene (Figure 13) and solid plate and plate with elliptical openings made of wood (Figure 14).

**Figure 15:**
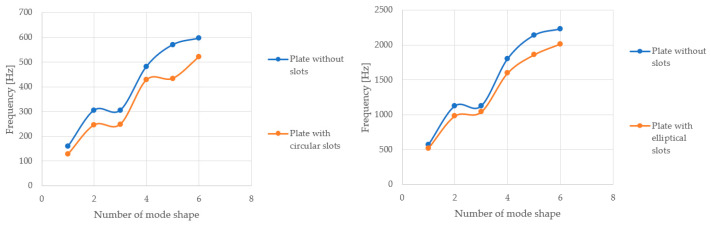
Charts depicting the frequency value of vibrations for a given mode shape at an airflow velocity of 1.5 m/s.

The shape of the successive vibration modes of the tested plates does not depend on the presence or distribution of the openings. Therefore, it can be deduced that the perforation of ceiling panels can be formed arbitrarily, as the plates subjected to airflow generated by air conditioners typically behave independently of the shape and size of the openings, their vibration modes take forms similar to solid plates. Comparing perforated plates to solid plates, a noticeable decrease in frequency values for successive vibration modes becomes apparent, caused by the reduced stiffness of the plates weakened by openings. The following graphs demonstrate that the nature of the frequency value increase for the compared plates remains unchanged (Figure 15).

**Figure 16:**
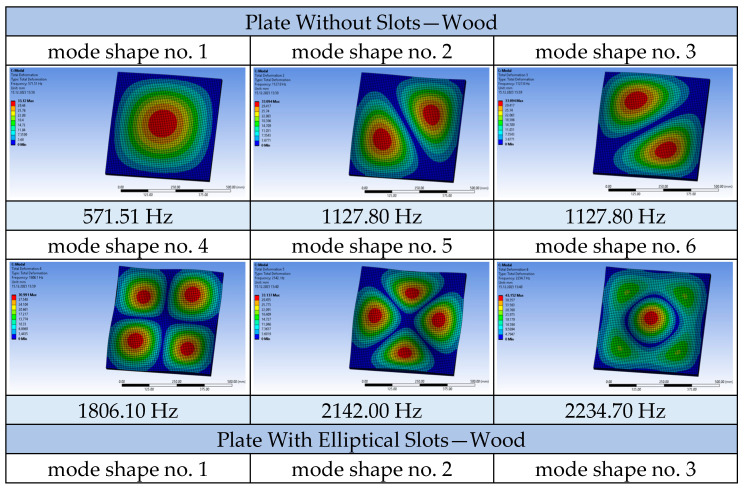

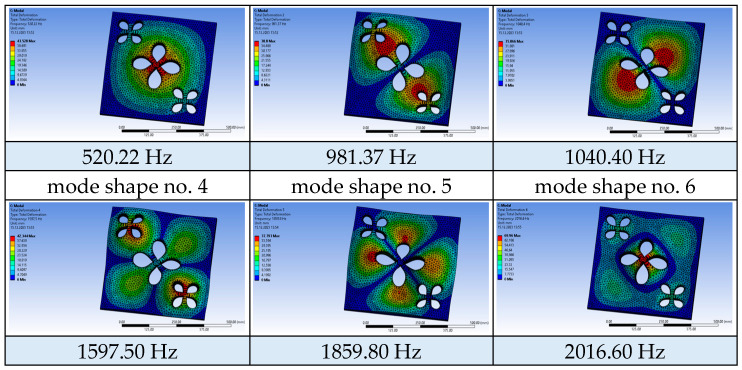
Mode shapes and frequencies for the plates made of wood at a flow velocity of 3.0 m/s.

**Figure 17:**
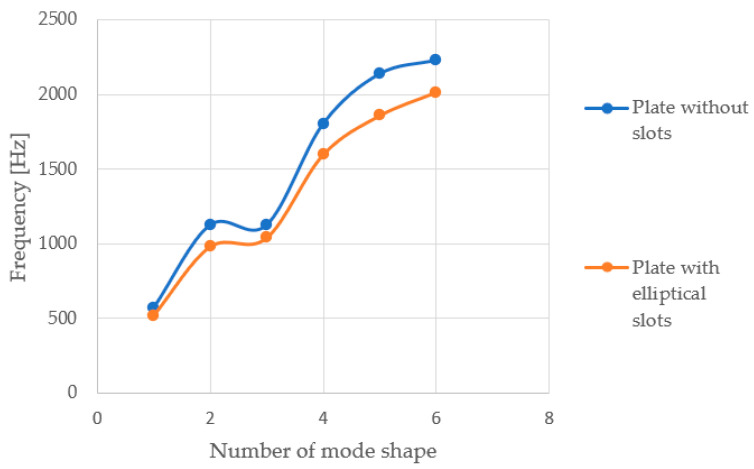
Charts depicting the frequency value of vibrations for a given mode shape at an airflow velocity of 3.0 m/s.

A comparison was also made between the solid plate and the plate with circular openings, which were subjected to airflow at twice the increased velocity, 3.0 m/s (cf. Figure 16 and Figure 17). Increased airflow velocity loading of the tested slabs does not significantly affect the appearance of the vibration modes. The nature of the frequency value increase for the compared solid plate and the plate with openings is analogous to the graphs obtained for calculations using an airflow speed of 1.5 m/s. Analysing the behaviour of all the tested plates in terms of modal analysis, it was observed that as the airflow velocity increases, the value of the vibration frequency of the system decreases slightly, which is in accordance with the principles of fluid mechanics. A tabular summary of the frequency results as a function of airflow velocity for one of the tested plates, geometry A2, is presented below (Table 3).

**Figure 18:**
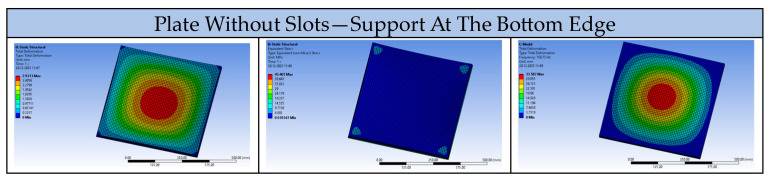

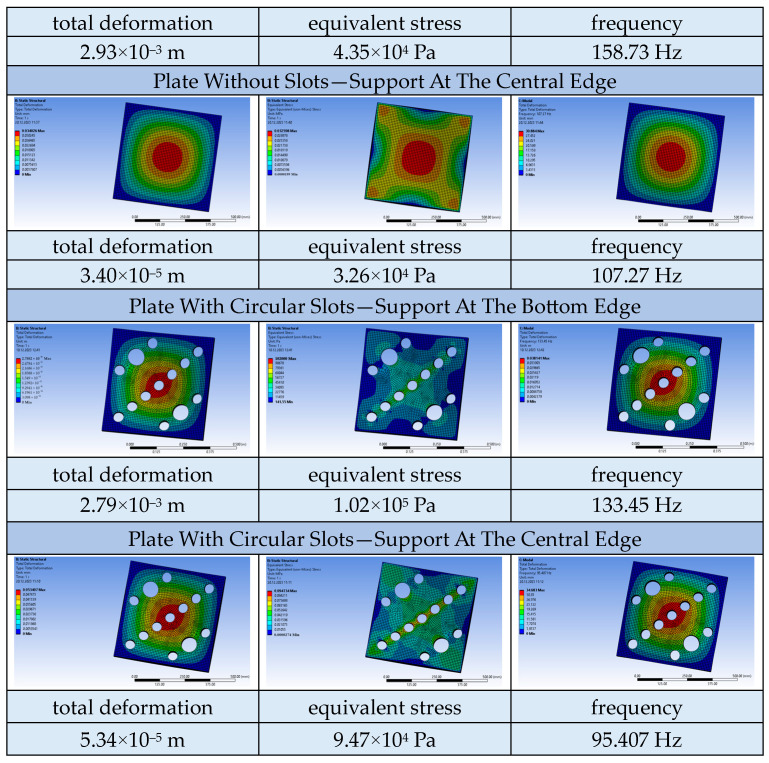
Comparison of deformation, stress, and vibration frequency of solid plates with support at the bottom edge and the middle plane.

The tested plates were modelled as elements supported at the bottom edge, as occurs in reality (the ceiling plates were mounted on a grid, resting on all the lower edges). For comparison, a model of a solid plate and a plate with circular perforations was created, with support applied in the middle plane. The results are presented in Figure 18. 

Based on the obtained results, it can be concluded that the location of the applied support has a significant impact on the results. The results were compared with the theoretical value calculated from the formula for the vibration frequency of solid plates made of polypropylene (PP), supported on all four edges, as follows:

(13) $$f=\alpha l^{-2}{\left[Et^{3}{\left(12m\left(1-\nu^{2}\right)\right)}^{-1}\right]}^{1/2}=200.68\;\mathrm{Hz},$$

where t is the thickness of the plate (0.02 m), E is Young’s modulus (1.461·10^9^ N/m^2^), ν is Poisson’s ratio (0.408), m is the mass of the plate per square meter (18.068 kg/m²), and the coefficient α, which depends on the plate’s support conditions, is calculated as follows:

(14) $$\alpha =1.57{\left(2.44+2.72\lambda^{2}+2.44\lambda^{4}\right)}^{1/2}=5.75,$$

where λ is the ratio of the plate’s side lengths. Therefore, the theoretical result is closer to the value obtained for the actual support of the plate at the lower edge.

**Figure 19:**
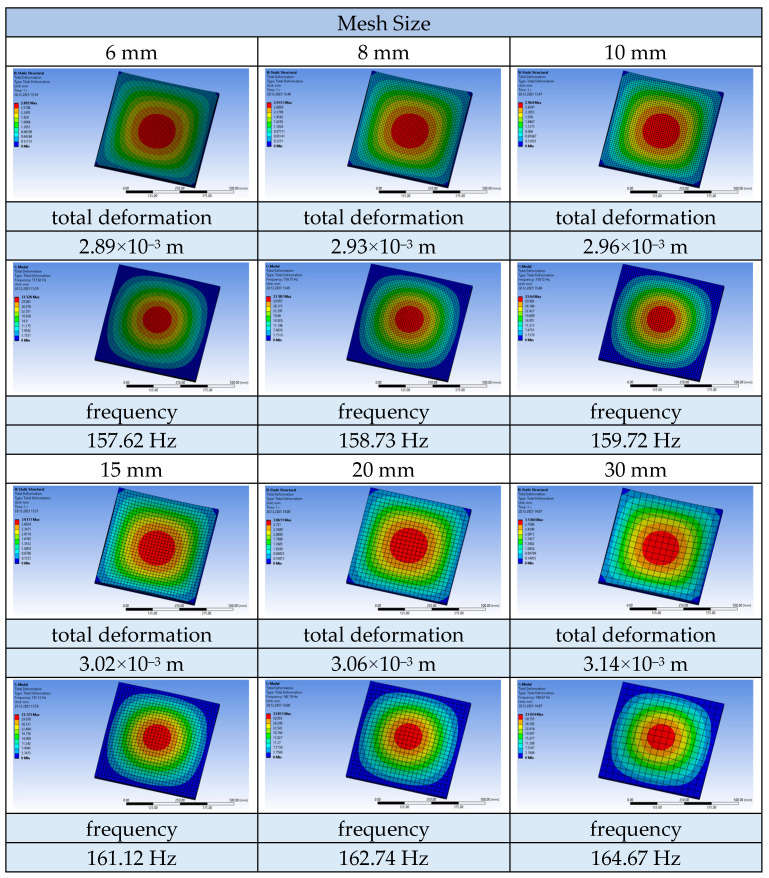
Comparison of deformation and vibration frequency for plates using different sizes of finite element mesh.

The impact of meshing on the results of the static and modal analysis of the tested plates was also examined. The deformation and vibration frequency results for the solid plate were compared for the following mesh element sizes: 6 mm, 8 mm, 10 mm, 15 mm, 20 mm, and 30 mm. The obtained results are shown in Figure 19.

**Figure 20:**
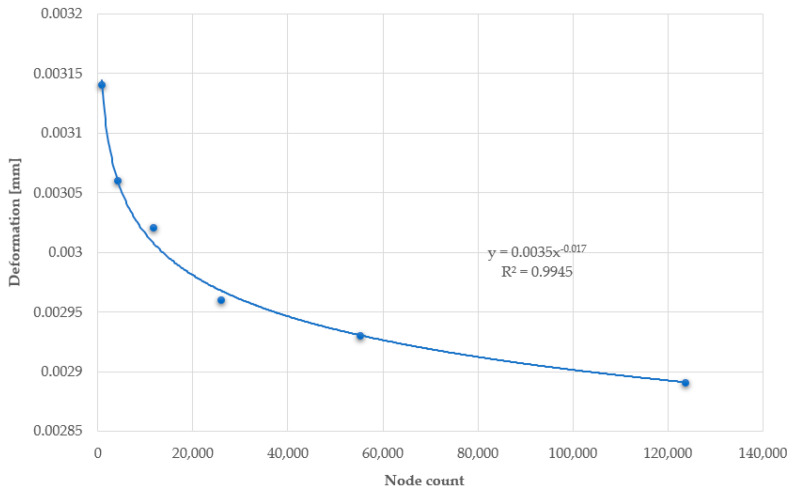
Chart of deformation values as a function of the number of nodes in the plate.

**Figure 21:**
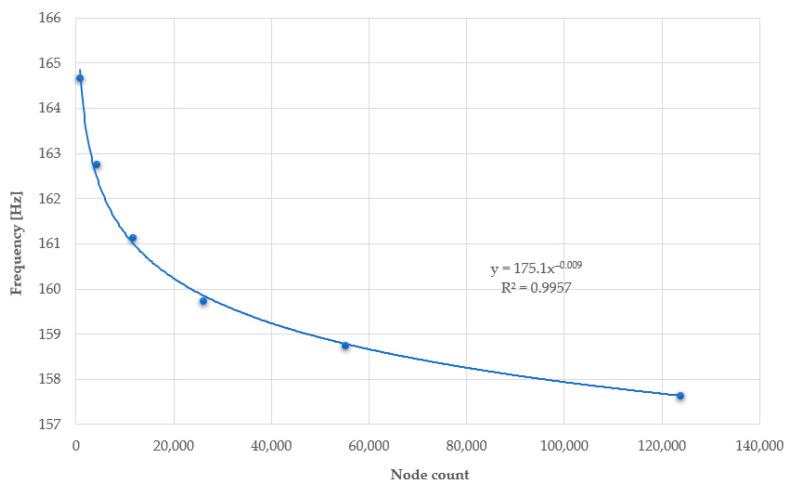
Chart of vibration frequency values as a function of the number of nodes in the plate.

Reducing the mesh size leads to the creation of a greater number of finite element mesh elements and, consequently, a greater number of nodes for which results are generated. Each of the studied plates was modelled in such a way as to have three finite elements in terms of thickness. The number of finite elements on the plates ranges between 800 and 130.000, depending on the number and surface area of the holes, as well as the mesh size used. Below are graphs depicting the relationship between deformation (Figure 20) and vibration frequency (Figure 21), as a function of the number of generated nodes.

The charts asymptotically show the correctly determined deformation and vibration frequency values. Too large finite element mesh elements lead to inaccuracies in the results. The relative error of the results when using a mesh size of 30 mm is 7.96% for deformations and 4.28% for vibration frequencies, compared to the results obtained using a mesh size of 6 mm. 

**Figure 22:**
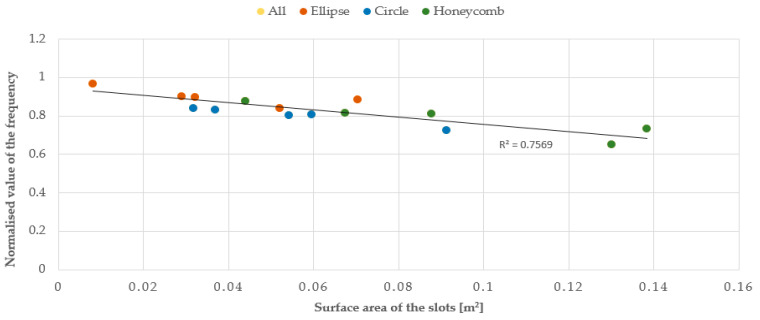
Chart of the relationship between vibration frequency and the total area of the openings, normalised in regard to the reference value for the solid plate.

**Figure 23:**
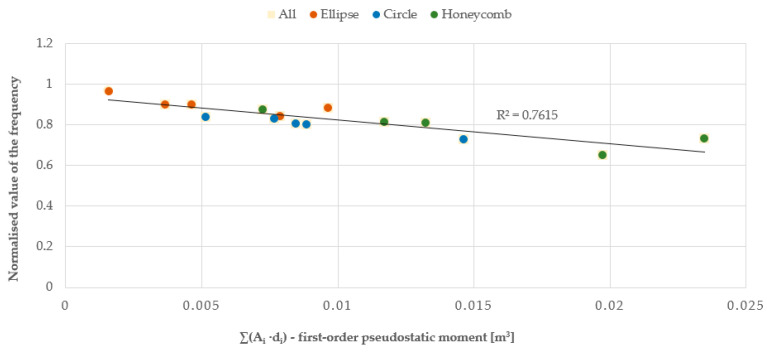
Chart of the relationship between vibration frequency and the first-order pseudostatic moment, normalized in regard to the reference value for the solid plate.

**Figure 24:**
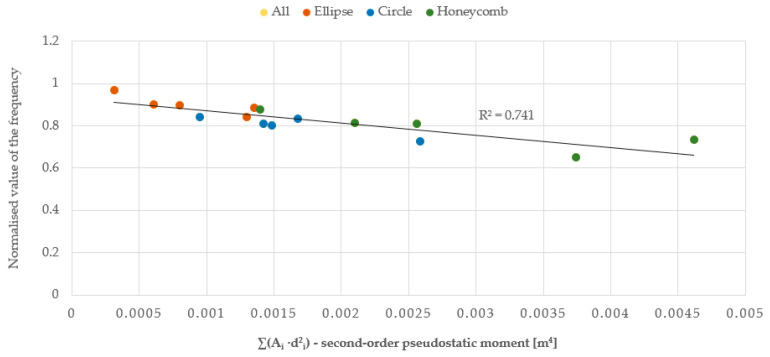
Chart of the relationship between vibration frequency and the second-order pseudostatic moment, normalised in regard to the reference value for the solid plate.

Subsequently, the vibration frequency values for the three series of plates were placed on composite graphs normalized in regard to a reference value, which was taken as the vibration frequency of the solid plate. Below are the dependencies of the vibration frequencies of the plates:

- From the total surface area of their openings (Figure 22);
- From the ‘pseudostatic’ first-order moment (Figure 23), i.e., the product of the total surface area of the individual openings (A_i_) and their distance from the centre of gravity of the plate (d_i_), expressed by formula:
  (15) $$\sum A_{i}d_{i},$$
- From the ‘pseudostatic’ second-order moment (Figure 24), i.e., the product of the total surface area of the individual openings (A_i_) and the square of their distance from the centre of gravity of the plate (d_i_), expressed by formula:
  (16) $$\sum A_{i}{\left(d_{i}\right)}^{2}.$$

**Figure 25:**
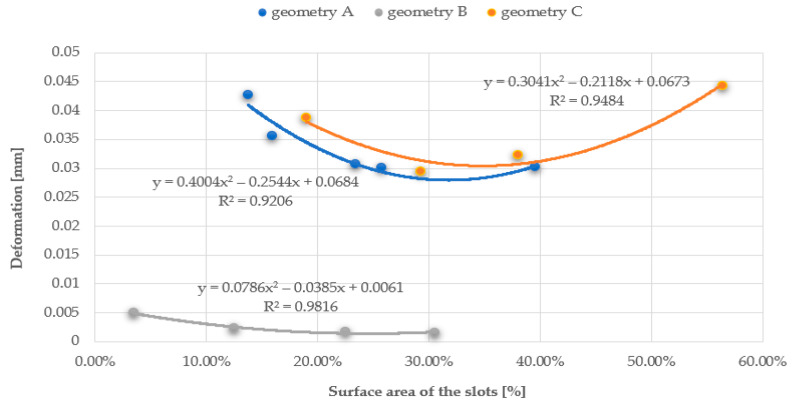
Chart of the relationship between deformations and the percentage surface area of the slots in relation to surface area of the entire plate.

**Figure 26:**
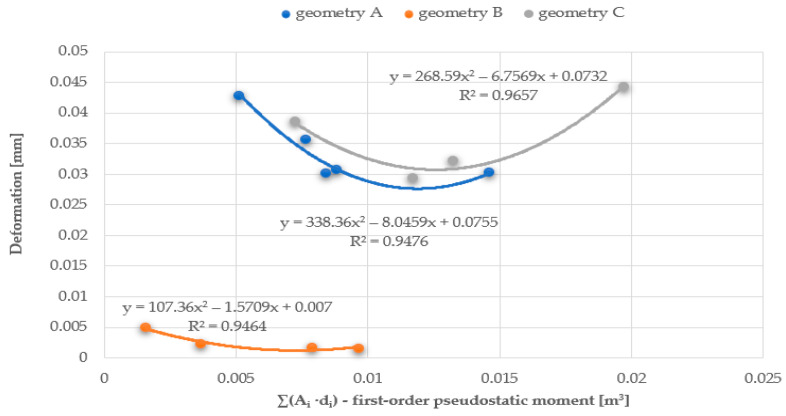
Chart of the relationship between deformations and the first-order ‘pseudostatic’ moment.

**Figure 27:**
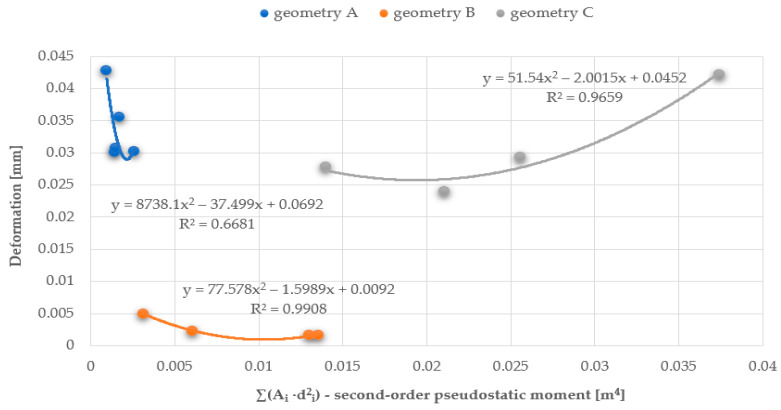
Chart of the relationship between deformations and the second-order ‘pseudostatic’ moment.

The relationship between the vibration frequency and the total area of the openings or the first and second-order ‘pseudostatic’ moments, which directly depend on the size and distribution of the openings, is approximately linear. As the area of the openings increases, a decrease in the vibration frequency of the plate is observed, as it allows the air to flow more freely, with less resistance. The best linear fit and, therefore, the smallest error calculated as the coefficient of determination R^2^, was obtained for the first-order ‘pseudostatic’ moment. Additionally, based on the results obtained from the static analysis of the plates conducted using the ANSYS software, graphs were created to show the relationship between the plate deformation and the total area of the openings, as well as the first and second-order ‘pseudostatic’ moments. The graphs were made for three series of plates and three airflow velocities. This article presents graphs for three different geometries and an airflow velocity of 3 m/s for series A (plate thickness 20 mm) and C plates (plate thickness 5 mm), and an airflow velocity of 1.5 m/s for series B plates (plate thickness 20 mm), cf. Figure 25, Figure 26 and Figure 27. 

The relationship between deformations and the number of openings in the plate forms parabolically, mainly convex parabolas, with a positive slope coefficient.

The best curve fitting to the results was obtained for the first-order ‘pseudostatic’ moment, as well as for higher airflow speeds (the best fit was observed for series B, as shown, with an airflow velocity of 3.0 m/s, achieving coefficient of determination R^2^ values of approximately 0.99); the influence of the pressure exerted on the plate is less decisive in these cases.

**Figure 28:**
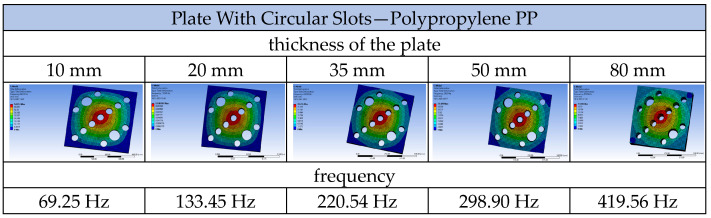
First mode shapes and their corresponding frequencies for plates with varying thicknesses.

**Figure 29:**
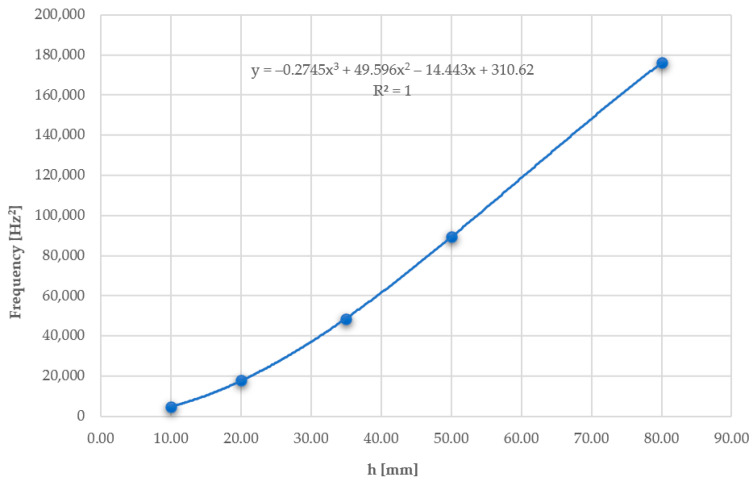
Chart of the relationship between the square of the frequency and the plate thickness.

The relationship between vibration frequency and plate thickness was also analysed. Plates with geometry A2 were introduced into the ANSYS program with five different thickness variants, 10, 20, 35, 50, and 80 mm. Each of them was subjected to airflow-induced loading at a specified velocity of 1.5 m/s. Figure 28 shows the obtained frequency results for the first mode of vibration. Meanwhile, Figure 29 depicts the relationship between the square of the frequency and the plate thickness.

Based on the above results, it can be observed that the vibration frequency increases parabolically with the increase in plate thickness.

**Figure 30:**
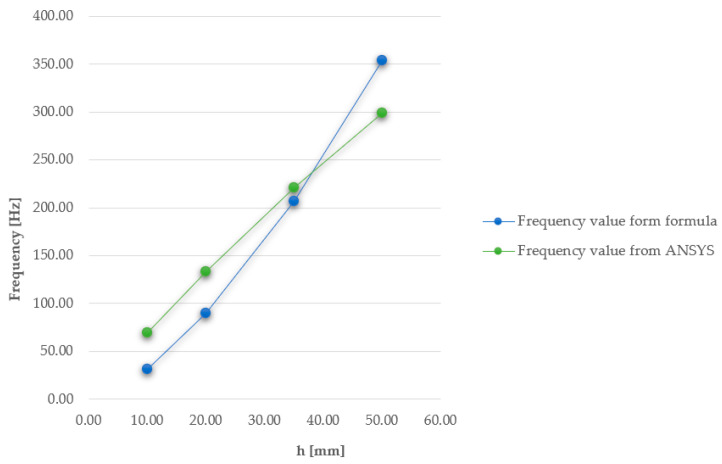
Comparative chart of vibration frequencies obtained using ANSYS software and from using the mathematical formula.

For thin plates with rigid support, there exists a relationship, described by the following formula:

(17) $$w\;≅\;h^{\frac{3}{2}},$$

where w is the vibration frequency and h is the plate thickness. Figure 30 shows the dependence of the vibration frequency values for a freely supported plate and its thickness, obtained using the ANSYS software, and the dependence of the vibration frequency values of a plate with rigid support and its thickness, obtained according to the above formula. This figure shows the effect of the fixing method on the dynamic behaviour of the plates. The frequency values vary by 6–52%, with respect to the higher frequency value.

## 4. Summary and Conclusions

In this study raster ceiling panels inspired by bionics were analysed numerically. This analysis was conducted in three distinct stages, consisting of a simulation of the airflow through the slab, a static analysis of the slab’s deformation and stresses, and a modal analysis to determine the dynamic properties.

When analysing airflow, the focus was primarily on determining the pressure exerted by the airflow on the surface of the panel, as well as its velocity and directions under the panel. This analysis showed a strong relationship between the pressure value and the field and location of the openings in the slab, as well as the air velocity at the inlet. For plates of the same thickness, made of the same material, the most commonly observed pressure values on the top surface varied between 2 Pa and 20 Pa, depending on the surface area of the holes in the plate and their distribution. The pressure distribution in the space between the panel and the ceiling was also shown to be strongly dependent on the area and location of the openings in the panel. Moreover, the air velocities and directions under the slab are influenced not only by the surface area of the openings, but also by the way they are distributed (uniformity).

The dynamic analysis of the panels primarily revealed that the mode shapes are independent of the presence or distribution of openings; the mode shapes of perforated panels take forms similar to those of solid panels. However, there are differences in the vibration frequency values between perforated and solid panels. The vibration frequencies of perforated panels, due to their lighter weight, are lower by an average of approx. 10–30% compared to unperforated panels, depending on the material, panel thickness, hole area, and mode shape. The airflow velocity also has a minor influence on the vibration frequencies, with an increase in airflow velocity resulting in a slight decrease in vibration frequency values of approximately 0.2–0.3% with an increase in velocity from 1.5 m/s to 10.0 m/s. The method of supporting the panel (lower edge or central surface) significantly affects the vibration frequencies. The frequency of both solid and perforated panels is approximately 30% lower when the panel support is modelled on the centre surface compared to the results obtained for modelling the support on the bottom edge. Additionally, the vibration frequency of the panels depends on the thickness, with the value increasing parabolically with increasing thickness.

On the other hand, the static analysis showed that the values of deformations and stresses on the panels are influenced by the method of support and the surface area of the openings. The relationship between deformations and the number of openings in the panels is parabolic.

The numerical models used are subject to many limitations. In the case of flow analysis, the numerical studies only take into account the influence of aerostatic pressure. The applied method of numerical analysis, therefore, ignores the effects related to self-excitation of lateral aerodynamic forces and flutter and spin excitation. These effects may be greater when increasing the airflow speed and decreasing the distance between individual holes.

It is also worth noting that the numerical models used assume ideal conditions of free support. In the case of real plates, such conditions are very difficult to implement due to the thickness of the plate and the risk of creating one-sided bonds during plate vibrations.

In further research, the authors intend to test real models to verify the obtained results. We plan to build a specialized test stand using various plates made of different materials. Both the airflow through the panel and the frequency of vibrations (free and dumped) will be tested.

## Figures and Tables

**Table 1:** Opening surface area and value of first- and second-order ‘pseudostatic’ moments.

	**A1**	**A2**	**A3**	**A4**	**A5**
Total slots area *A* [m^2^]	0.05938	0.031647	0.03676	0.091102	0.054039
First pseudostatic moment ∑A_i_d_i_ [m^3^]	0.00843522	0.005140504	0.007669144	0.014613606	0.008837521
Second pseudostatic moment ∑A_i_d_i_^2^ [m^4^]	0.001424596	0.000947058	0.001678304	0.002582505	0.001485135
	**B1**	**B2**	**B3**	**B4**	**B5**
Total slots area *A* [m^2^]	0.051954722	0.070359903	0.00801954	0.032078151	0.028817328
First pseudostatic moment ∑A_i_d_i_ [m^3^]	0.007881531	0.009649025	0.001570867	0.004622463	0.00365624
Second pseudostatic moment ∑A_i_d_i_^2^ [m^4^]	0.001296856	0.001354151	0.000312921	0.000801851	0.000605092
	**C1**	**C2**	**C3**	**C4**	**C5**
Total slots area *A* [m^2^]	0.138251	0.129905	0.06734	0.043771	0.087542
First pseudostatic moment ∑A_i_d_i_ [m^3^]	0.02345967	0.019714383	0.011703692	0.007226255	0.013220189
Second pseudostatic moment ∑A_i_d_i_^2^ [m^4^]	0.004615813	0.003739818	0.002102095	0.001397285	0.002557995

**Table 2:** Material properties and thickness of the tested panels.

**Series A—Plates with Circular Slots**				
Material	Plate thickness [mm]	Density [kg/m^3^]	Young’s modulus [MPa]	Poisson’s ratio [-]
Polypropylene PP	20	903.4	1461	0.408
Polypropylene PP	50	903.4	1461	0.408
Polypropylene PP	80	903.4	1461	0.408
**Series B—Plates with Elliptical Slots**				
Material	Plate thickness [mm]	Density [kg/m^3^]	Young’s modulus [MPa]	Poisson’s ratio [-]
Wood	20	935.7	22,780	0.3742
Wood	50	935.7	22,780	0.3742
Wood	80	935.7	22,780	0.3742
**Series C—Plates with Hexagonal Slots**				
Material	Plate thickness [mm]	Density [kg/m^3^]	Young’s modulus [MPa]	Poisson’s ratio [-]
Aluminium	2	2770	71,000	0.33
Aluminium	5	2770	71,000	0.33
Aluminium	8	2770	71,000	0.33

**Table 3:** Vibration frequencies of the plate with circular openings (geometry A2).

Plate with Circular Slots—Polypropylene PP—Geometry A2			
Velocity of Airflow	1.5 m/s	3.0 m/s	10.0 m/s
Frequency for 1st mode shape:	133.45 Hz	133.42 Hz	133.05 Hz
Frequency for 2nd mode shape:	263.40 Hz	263.36 Hz	262.90 Hz
Frequency for 3rd mode shape:	269.43 Hz	269.40 Hz	269.00 Hz
Frequency for 4th mode shape:	430.87 Hz	430.84 Hz	430.43 Hz
Frequency for 5th mode shape:	496.17 Hz	496.14 Hz	495.67 Hz
Frequency for 6th mode shape:	534.24 Hz	534.21 Hz	533.77 Hz

## Data Availability

Data are unavailable due to privacy.
